# Machine learning models for the prediction of hydrogen solubility in aqueous systems

**DOI:** 10.1038/s41598-025-16289-7

**Published:** 2025-08-26

**Authors:** Mehdi Maleki, Ali Akbari, Yousef Kazemzadeh, Ali Ranjbar

**Affiliations:** Department of Petroleum Engineering, Faculty of Petroleum, Gas, and Petrochemical Engineering, Persian Gulf University, Bushehr, Iran

**Keywords:** Hydrogen storage, Solubility, Saline aquifers, Machine learning, Reservoir optimization, Fluid dynamics, Engineering, Chemical engineering, Energy infrastructure

## Abstract

Hydrogen storage is integral to reducing CO_2_ emissions, particularly in the oil and gas industry. However, a primary challenge involves the solubility of hydrogen in subsurface environments, particularly saline aquifers. The dissolution of hydrogen in saline water can impact the efficiency and stability of storage reservoirs, necessitating detailed studies of fluid dynamics in such settings. Beyond its role as a clean energy carrier and precursor for synthetic fuels and chemicals, understanding hydrogen’s solubility in subsurface conditions can significantly enhance storage technologies. When hydrogen solubility is high, it can reduce reservoir pressure and alter the chemical composition of the storage medium, undermining process efficiency. Machine learning techniques have gained prominence in predicting physical and chemical properties across various systems. One of the most complex challenges in hydrogen storage is predicting its solubility in saline water, influenced by factors such as pressure, temperature, and salinity. Machine learning models offer substantial promise in improving hydrogen storage by identifying intricate, nonlinear relationships among these parameters. This study uses machine learning algorithms to predict hydrogen solubility in saline aquifers, employing techniques such as Bayesian inference, linear regression, random forest, artificial neural networks (ANN), support vector machines (SVM), and least squares boosting (LSBoost). Trained on experimental data and numerical simulations, these models provide precise predictions of hydrogen solubility, which is strongly influenced by pressure, temperature, and salinity, under a wide range of thermodynamic conditions. Among these methods, RF outperformed the others, achieving an R^2^ of 0.9810 for test data and 0.9915 for training data, with RMSE values of 0.048 and 0.032, respectively. These findings emphasize the potential of machine learning to significantly optimize hydrogen storage and reservoir management in saline aquifers.

## Introduction

A primary factor driving the increasing focus on hydrogen storage within the oil industry is its significant potential to reduce carbon dioxide emissions^1^. Hydrogen plays a vital role in refining by aiding in the desulfurization of fuels, ensuring compliance with rigorous environmental standards^2^.

Furthermore, replacing fossil fuels with hydrogen in the thermal units of refineries and petrochemical plants could lead to a substantial decrease in greenhouse gas emissions, thus supporting broader decarbonization goals^3^. In addition to being a clean energy carrier, hydrogen is an essential raw material in the production of synthetic fuels and valuable chemical products. As the demand for low-carbon fuels and environmentally responsible products rises, hydrogen storage and the advancement of related technologies offer a distinct competitive advantage for oil and gas companies, promoting innovation and enhanced market positioning.

Operationally, storing hydrogen in depleted oil fields and saline aquifers offers a promising opportunity to optimize resource utilization. Injecting hydrogen into these subsurface reservoirs not only helps manage reservoir pressure but also contributes to enhanced oil recovery (EOR) through complex fluid dynamics. This strategy not only improves economic efficiency but also reduces the environmental risks associated with aging reservoirs^4–11^.

Beyond operational gains, the long-term stability of hydrogen storage requires careful analysis of subsurface interactions to maintain the integrity of geological formations^12–14^. To ensure the durability of storage systems, advanced monitoring and predictive modeling techniques must be utilized to evaluate reservoir behavior and mitigate risks related to hydrogen migration or reactivity^15–18^.

The storage of hydrogen has emerged as a pivotal strategy for mitigating dependence on fossil fuels and attenuating greenhouse gas emissions. Nevertheless, the efficient and secure containment of this highly volatile and lightweight element remains a formidable challenge, particularly when implemented within natural formations such as saline aquifers or subsurface geological reservoirs. A critical dimension of this endeavor entails the elucidation of hydrogen solubility dynamics in saline aqueous environments and the resultant environmental ramifications, necessitating rigorous and multidisciplinary investigations^19–23^.

The concept of solubility pertains to the maximum quantity of a substance that can dissolve in a given solvent, influenced by parameters such as temperature and pressure. The general equation for calculating solubility is expressed as^24^:

1 $$\:S=\frac{m}{V}$$

$\:S$ represents the solubility, denoting the quantity of solute that has dissolved in the solvent, $\:m$ is the mass of the solute and $\:V$ is the volume of the solvent^25,26^.

In the context of hydrogen storage, one of the most critical factors to consider is the solubility of hydrogen in saline aqueous environments and the subsequent implications for both storage mechanisms and environmental sustainability. Although hydrogen typically exhibits limited solubility in water under standard conditions, under specific circumstances—such as elevated pressures and reduced temperatures—its dissolution in saline water is plausible. To quantify the solubility of hydrogen in these environments, models such as Henry’s Law are frequently employed, offering a rigorous framework for experimental validation^27–30^.

The Henry’s Law equation is formulated as:

2 $$\:C={K}_{H}\:.\:\:$$

$\:C$ is the concentration of the dissolved gas in the liquid phase, $\:{K}_{H}\:$is the Henry’s law constant, indicative of the solubility of a particular gas in a given solvent and $\:P$ refers to the partial pressure exerted by the gas. When considering saline aquifers, the presence of dissolved ions can significantly alter the solubility of hydrogen, with increased salinity potentially reducing hydrogen’s capacity to dissolve in the solution^31–33^. This dynamic is of paramount importance when assessing the viability of hydrogen storage in subterranean reservoirs, where the solubility of hydrogen in such geological formations may give rise to environmental concerns, including the depletion of dissolved oxygen and the emergence of anaerobic conditions, which could profoundly disrupt local ecosystems^34–36^.

In recent years, machine learning techniques have gained prominence as invaluable tools for forecasting a wide range of physical and chemical properties within various systems. One particularly intricate challenge in the field of hydrogen storage is accurately predicting its solubility in saline aquatic environments^37–39^. Hydrogen solubility in these media is governed by a multitude of factors, including pressure, temperature, and salt concentration^40–43^. Thus, the application of advanced machine learning models to analyze and predict this characteristic can substantially enhance the efficiency and effectiveness of hydrogen storage methodologies in subterranean reservoirs. Machine learning models are adept at discerning complex, nonlinear relationships between parameters such as temperature, pressure, salt concentration, and hydrogen solubility^44–46^. A widely adopted approach in this context is the use of regression algorithms, which enable the prediction of hydrogen solubility across diverse conditions by leveraging empirical data. These models prove especially invaluable in scenarios where experimental data is sparse or limited^47^.

The integration of experimental data and computational simulations as inputs to machine learning models significantly bolsters the precision of hydrogen solubility predictions in saline environments^48^. These data can be derived from laboratory experiments or sophisticated numerical simulations, both of which provide a rich foundation for machine learning models to identify trends and solubility dynamics. This approach not only reduces the financial and temporal costs of experimental research but also accelerates the development of innovative technologies in the field of hydrogen storage^1^. Ultimately, the insights derived from these predictive models serve as a crucial asset for large-scale decision-making and real-world applications. Enhanced accuracy in predicting hydrogen solubility is particularly advantageous for optimizing storage strategies in subsurface reservoirs and saline aquifers. By harnessing the power of machine learning, researchers and industry professionals can attain a deeper comprehension of hydrogen’s behavior under varying conditions, thereby advancing the development of optimal, sustainable storage solutions for this eco-friendly energy carrier^49–53^.

In 2024, several studies explored machine learning approaches to predict hydrogen solubility in aqueous environments, a critical factor for underground hydrogen storage (UHS). Dehghani et al.^54^ developed and evaluated nine models, with LSBoost achieving the highest accuracy (R^2^ = 0.9997, RMSE = 4.18E-03), demonstrating strong predictive capability. Longe et al.^55^ employed deep learning, integrating CNN and LSTM with optimization algorithms, identifying CNN-GO as the most effective model (RMSE = 0.00006 for training). Their analysis confirmed pressure as the dominant factor influencing solubility. Altalbawy et al.^56^ focused on hydrogen/methane mixtures, using hybrid models such as LSSVM-GA and LSSVM-CSA, which outperformed others in accuracy. Pressure and hydrogen mole fraction were found to be key solubility determinants. Mwakipunda et al.^57^ introduced PSO-MERF, surpassing traditional models with an R of 0.9982 and RMSE of 0.0015, while emphasizing salinity as the most influential factor. Collectively, these studies underscore the effectiveness of machine learning in enhancing solubility predictions, optimizing UHS systems, and ensuring storage efficiency.

In 2023, Thanh et al. and Nakhaei-Kohani et al. conducted studies on hydrogen solubility prediction, focusing on aqueous systems with varying salinity and ionic liquids (ILs), respectively. Thanh et al.^23^ utilized four machine learning models, with adaptive gradient boosting achieving the highest accuracy (R^2^ = 0.994, RMSE = 0.018, MAE = 0.006) using 255 data points. Nakhaei-Kohani et al.^58^ examined 13 ILs across 580 data points, employing models like Random Forest, AdaBoost-SVR, DBN, and MARS, with DBN outperforming others (RMSE = 0.00106 and 0.00066). Sensitivity analysis highlighted pressure and the –CH_3_ group as key factors. Both studies demonstrate the effectiveness of machine learning in improving hydrogen storage predictions and advancing energy storage solutions.

In a 2022 study, Ansari et al.^40^ undertook a sophisticated exploration of hydrogen solubility in aqueous solutions, critical for the optimization of underground hydrogen storage (UHS). The study aimed to compare the efficacy of advanced machine learning models against traditional equations of state (EoSs) for accurate solubility prediction under varying conditions of temperature and pressure. The authors employed Radial Basis Function (RBF) and Least Squares Support Vector Machine (LSSVM) models, optimized through metaheuristic algorithms, including biogeography-based optimization (BBO), cultural algorithm (CA), imperialist competitive algorithm (ICA), and teaching-learning-based optimization (TLBO). Among these, the RBF + CA model emerged as the most precise, exhibiting a root mean square error of 0.000176 and a correlation coefficient of 0.9792. When compared with four renowned EoSs—Soave-Redlich-Kwong (SRK), Peng-Robinson (PR), Redlich-Kwong (RK), and Zudkevitch-Joffe (ZJ)—the SRK equation demonstrated the strongest performance, yet the machine learning models surpassed them in terms of accuracy. The findings revealed that pressure and temperature were the most influential factors affecting hydrogen solubility. The RBF + CA model proved to be a robust tool for accurately predicting solubility in both pure and saline water under typical underground storage conditions. This study highlights the promising role of machine learning in enhancing the precision and efficiency of hydrogen storage technologies.

In a 2021 study, Mohammadi et al.^26^ investigated hydrogen solubility in hydrocarbon fuels, a critical parameter for various industrial applications, particularly in petroleum refineries and coal processing plants. The research aimed to develop reliable models for predicting hydrogen solubility across diverse hydrocarbon fuels and feedstocks, utilizing advanced machine learning methods. The authors employed four novel machine learning techniques—Extreme Gradient Boosting (XGBoost), Multi-layer Perceptron (MLP) trained with the Levenberg–Marquardt algorithm, Adaptive Boosting Support Vector Regression (AdaBoost-SVR), and LiteMORT, a memory-efficient gradient boosting tree system. The study utilized a dataset comprising 445 experimental data points covering 17 different hydrocarbon fuels, including petroleum fractions, refinery products, and coal liquids. Key input parameters included temperature, pressure, density, molecular weight, and the weight% of carbon and hydrogen in the fuels. Among the models, XGBoost demonstrated the highest accuracy, with a mean absolute percentage error of just 1.41% and a coefficient of determination (R^2^) of 0.9998. Additionally, seven equations of state (EoSs) were applied to compare solubility predictions, with the 2- and 3-parameter Soave-Redlich-Kwong (SRK) EoS providing the most accurate results. Sensitivity analysis revealed that pressure was the most influential factor on hydrogen solubility, followed by temperature and hydrogen weight%. The findings highlight the XGBoost model’s potential as a reliable tool for estimating hydrogen solubility in hydrocarbon fuels, which can optimize industrial processes related to hydrogen storage and fuel utilization.

The study of hydrogen storage in subsurface reservoirs, particularly within saline aquifers, holds increasing significance in the context of global efforts to mitigate greenhouse gas emissions and transition towards sustainable energy systems. As a clean energy carrier, hydrogen has the potential to play a pivotal role in reducing dependence on fossil fuels and combating environmental pollution.

However, the efficient and safe storage of this gas in subsurface formations, especially in saline and aqueous environments, presents a variety of challenges, including the accurate prediction of hydrogen solubility in these settings. Research has demonstrated that factors such as pressure, temperature, and salinity can significantly impact the solubility of hydrogen in saline water. Consequently, the development of precise predictive models is essential to enhance hydrogen storage processes.

In recent years, machine learning models have emerged as powerful tools for predicting the complex physical and chemical properties of such systems, offering an innovative approach to understanding and forecasting hydrogen solubility in aqueous environments. These models are capable of simulating the nonlinear relationships between variables such as pressure, temperature, and salinity, thus providing accurate predictions under various conditions.

Additionally, leveraging experimental data and numerical simulations as inputs for machine learning models enhances the precision of these predictions while simultaneously reducing the cost and time required for physical experiments. Ultimately, such models enable researchers and industry professionals to design more efficient and optimized hydrogen storage strategies in subsurface environments.

**Table 1:** Skimming on literature review.

Authors	Year	Objective	Methods/models	Numerical results	Theoretical conclusion
Thanh et al.^23^	2023	Predict hydrogen solubility in aqueous systems of varying salinity.	Adaptive Gradient Boosting, Gradient Boosting, Random Forest, Extreme Gradient Boosting	R^2^ = 0.994, MAE = 0.006, RMSE = 0.018; 18 outliers identified using Williams plot	Adaptive Gradient Boosting is highly reliable for hydrogen storage prediction.
Dehghani et al.^54^	2024	Develop ML models for predicting hydrogen solubility in saline aquifers.	LSBoost, ANN, CatBoost, SVM with Bayesian Optimization	R^2^ = 0.9997, RMSE = 4.18E-03, maximum residual error = − 0.0252	Pressure correlates positively with solubility; LSBoost offers robust predictive capability.
Longe et al.^55^	2024	Develop reliable models for H_2_solubility prediction in saline systems.	CNN, LSTM, Growth Optimization (GO), Grey Wolf Optimization (GWO)	RMSE (Training) = 0.00006, RMSE (Testing) = 0.00021	CNN-GO captures nonlinear relationships; Pressure is the most influential parameter.
Altalbawy et al.^56^	2024	Model solubility of hydrogen/methane mixtures in brine.	ANFIS, LSSVM optimized with PSO, GA, CSA	LSSVM-GA and LSSVM-CSA achieved the highest R^2^ and lowest AARE%	Machine learning models are valuable for predicting solubility in complex gas mixtures.
Ansari et al.^40^	2022	Compare ML models and EoSs for predicting hydrogen solubility.	RBF, LSSVM optimized using BBO, CA, ICA, TLBO; Soave-Redlich-Kwong (SRK), Peng-Robinson (PR), RK, ZJ EoS	RBF + CA RMSE = 0.000176, R^2^ = 0.9792	ML models outperformed traditional EoSs; Pressure and temperature are key factors.
Mohammadi et al.^26^	2021	Develop predictive models for hydrogen solubility in hydrocarbon fuels.	XGBoost, MLP, AdaBoost-SVR, LiteMORT	XGBoost MAPE = 1.41%, R^2^ = 0.9998	XGBoost is highly accurate for hydrogen solubility prediction in hydrocarbon systems.
Nakhaei-Kohani et al.^58^	2023	Predict hydrogen solubility in ionic liquids.	RF, AdaBoost-SVR, DBN, MARS	DBN RMSE = 0.00106 (Approach 1), 0.00066 (Approach 2)	Pressure and –CH3 group significantly affect hydrogen solubility in ionic liquids.
Mwakipunda et al.^57^	2024	Enhance hydrogen solubility predictions for UHS applications.	PSO-MERF, XGBoost, KNN, RF, EOS models	PSO-MERF *R* = 0.9982, RMSE = 0.0015, MAE = 0.00091	PSO-MERF shows superior accuracy and efficiency; Salinity is a key factor in UHS operations.

According to Table 1 the reviewed studies collectively underscore the transformative role of machine learning in advancing the predictive modeling of hydrogen solubility in aqueous systems, particularly for underground hydrogen storage (UHS). By leveraging sophisticated algorithms and optimization techniques, researchers have significantly enhanced the accuracy and reliability of solubility predictions under diverse temperature, pressure, and salinity conditions. Hydrogen solubility is significantly influenced by pressure and temperature. These thermodynamic variables control the dissolution capacity of hydrogen in saline water, making them essential parameters in predictive modeling. The findings consistently highlight the dominant influence of parameters such as pressure and salinity on hydrogen solubility, while emphasizing the superiority of models like LSBoost, CNN-GO, and DBN in capturing complex nonlinear relationships. These advancements not only address critical predictive challenges but also lay a solid foundation for the development of more efficient, cost-effective, and sustainable hydrogen storage technologies. The insights gained from these studies will be instrumental in fostering the broader adoption of hydrogen as a clean and renewable energy carrier, supporting the transition toward a more sustainable energy future.

In this study, delve into the application of machine learning models for predicting hydrogen solubility in saline aquifers. The models discussed herein include algorithms such as the bayesian method, Linear Regression, Random Forest, Artificial Neural Networks (ANN), Support Vector Machines (SVMs) and Least Squares Boosting (LSBoost). These algorithms are adept at analyzing experimental and numerical simulation data, offering enhanced predictive capabilities for hydrogen solubility under varying temperature and pressure conditions. Subsequently, present a comparative analysis of the accuracy and performance of different machine learning models, exploring their potential applications in hydrogen storage within saline aquifers and subsurface reservoirs. The outcomes of this research could provide valuable insights into advancing hydrogen storage technologies and optimizing subsurface storage processes, ultimately contributing to more sustainable and efficient energy systems.

## Methods

### Data collection & processing

**Fig. 1:**
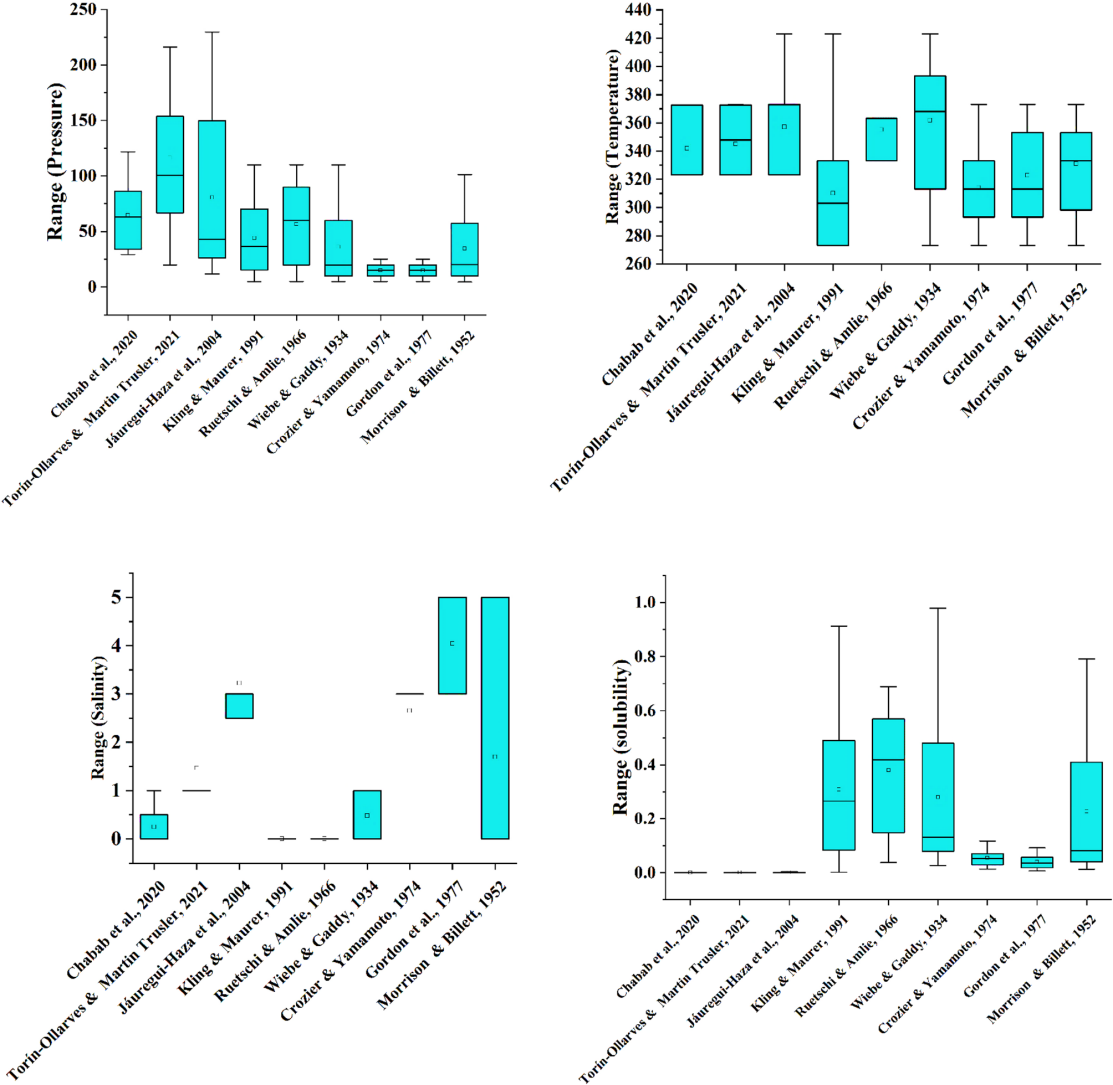
The range of input data used from previous studies available in various databases.

In this study, the data required to develop high-accuracy and efficient machine learning models were collected from previous research available in various databases. The aim of this process was to ensure comprehensiveness and extensive coverage of information. Figure 1 presents the details of the extracted data, and box plots were used to analyze data distribution and identify key features. These plots provide insights into the mean and data dispersion.

The data were selected to cover a wide range of physical and chemical variables, including temperature, pressure, and salinity. Initially, a total of 1020 data points were collected from various literature sources. Following outlier detection and removal using the Gaussian method, the final dataset comprised 992 valid samples, which were subsequently used for machine learning model development. Additionally, the data include information on both pure and saline water, which enhances the diversity and applicability of the models. The broad range of data ensures that machine learning models can predict and analyze complex scenarios effectively. The necessary statistical information is detailed in Table 2. The collected dataset was compiled from the following literature sources: Chabab et al.^59^, Ollarves & Trusler^60^, Haza et al.^61^, Kling & Maurer^62^, Ruetschi & Amlie^63^, Wiebe & Gaddy^64^, Crozier & Yamamoto^65^, Gordon et al.^66^, and Morrison & Billett^67^.

The final dataset of 992 data points was randomly divided into training and testing subsets using varying ratios ranging from 10 to 90%. For each ratio, ten independent random splits were performed, and the average performance metrics were reported.

**Table 2:** Data statistics.

		Chabab et al.^59^	Ollarves & Trusler.^60^	Haza et al.^61^	Kling & Maurer^62^	Ruetschi & Amlie^63^	Wiebe & Gaddy^64^	Crozier & Yamamoto^65^	Gordon et al.^66^	Morrison & Billett^67^
Pressure (bar)	max	121.706	216.205	229.720	110.000	110.000	110.000	25.000	25.000	101.400
	min	29.272	19.884	11.640	5.000	5.000	5.000	5.000	5.000	4.600
	range	92.434	196.321	218.080	105.000	105.000	105.000	20.000	20.000	96.800
	median	63.141	100.832	42.640	36.485	60.000	20.000	15.000	15.000	20.100
	Q1	32.398	63.860	25.408	15.2	20	10	10	10	10
	Q3	89.911	162.142	150.249	70	90	60	20	20	59.05
	mean	64.748	116.499	80.585	44.103	56.667	36.111	15.000	14.783	34.787
	variance	934.465	3612.946	4957.734	1014.971	1145.556	1116.358	45.652	53.214	937.805
	skewness	0.582	0.120	0.906	0.617	− 0.096	1.081	0	0.081	0.979
	kurtosis	− 0.469	− 1.087	− 0.681	− 0.826	− 1.436	− 0.242	− 1.195	− 1.414	− 0.397
Temperature (K)	max	372.730	372.780	423.150	423.150	363.150	423.150	373.150	373.150	373.150
	min	323.180	323.180	323.150	273.150	333.150	273.150	273.150	273.150	273.150
	range	49.550	49.600	100.000	150.000	30.000	150.000	100.000	100.000	100.000
	median	323.205	347.900	372.745	303.150	363.150	368.150	313.150	313.150	333.150
	Q1	323.183	323.21	323.19	273.15	333.15	313.15	293.15	293.15	298.15
	Q3	372.718	372.725	373.15	333.15	363.15	400.65	333.15	353.15	353.15
	mean	341.765	345.041	357.144	310.150	355.150	361.854	314.020	322.715	331.075
	variance	574.927	424.604	992.659	1546	176	2611.283	1129.679	1491.115	1098.523
	skewness	0.644	0.248	0.415	1.616	− 1.176	− 0.345	0.574	0.035	− 0.346
	kurtosis	− 2.240	− 1.626	− 0.513	2.894	− 0.734	− 1.242	− 0.702	− 1.720	− 0.999
Salinity (% by weight)	max	1.000	3.000	5.000	–	–	1.000	3.000	5.000	5.000
	min	0.000	1.000	2.500	–	–	0.000	1.000	3.000	0.000
	range	1.000	2.000	2.500	–	–	1.000	2.000	2.000	5.000
	median	0.000	1.000	3.000	–	–	0.000	3.000	5.000	0.000
	Q1	0	1	2.5	–	–	0	3	3	0
	Q3	0.75	2	3.5	–	–	1	3	5	5
	mean	0.250	1.471	3.227	–	–	0.481	2.652	4.043	1.698
	variance	0.188	0.720	0.971	–	–	0.250	0.575	0.998	5.607
	skewness	1.440	1.372	1.221	–	–	0.076	− 1.843	− 0.093	0.697
	kurtosis	0	− 0.149	-0.284	–	–	− 2.072	1.522	− 2.190	− 1.575
Hydrogen solubility (mole%)	max	0.002	0.002	0.005	0.913	0.690	0.979	0.146	0.093	0.792
	min	0.000	0.000	0.0001	0.002	0.038	0.027	0.013	0.007	0.013
	range	0.001	0.002	0.004	0.911	0.652	0.952	0.133	0.086	0.779
	median	0.001	0.001	0.001	0.265	0.420	0.131	0.052	0.037	0.082
	Q1	0.0004	0.0005	0.0006	0.078	0.148	0.074	0.030	0.019	0.041
	Q3	0.0013	0.0016	0.0022	0.500	0.571	0.499	0.071	0.057	0.413
	mean	0.001	0.001	0.002	0.309	0.380	0.281	0.055	0.040	0.228
	variance	1.836E-7	3.714E-07	1.514E-6	0.066	0.045	0.079	0.001	0.0007	0.054
	skewness	0.521	0.264	1.136	0.635	− 0.245	1.032	1.048	0.723	0.923
	kurtosis	− 1.182	− 1.096	0.611	− 0.637	− 1.441	− 0.378	1.380	− 0.683	− 0.589

For data analysis, various tools such as histograms, heat maps, box plots, and violin plots are widely used. A histogram displays the distribution of a numerical variable by dividing the data into intervals and showing the frequency of each interval as bars. A heat map uses colors to represent the intensity or value of data in a two-dimensional matrix, and it is useful for analyzing correlation matrices and multidimensional data. A box plot provides a summary of data distribution by displaying the median, quartiles, and interquartile range, and it is suitable for identifying outliers and comparing distributions. Meanwhile, a violin plot, which combines a box plot and a density plot, not only shows the median and quartiles but also illustrates the shape of the data distribution using a density curve, making it useful for more detailed analysis and identifying the multimodality of distributions.

**Fig. 2:**
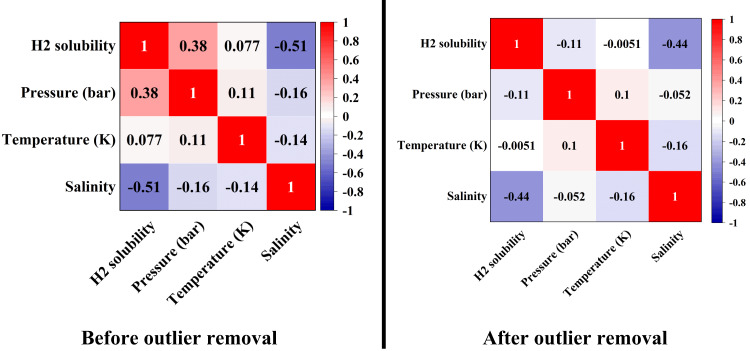
Heat maps.

**Fig. 3:**
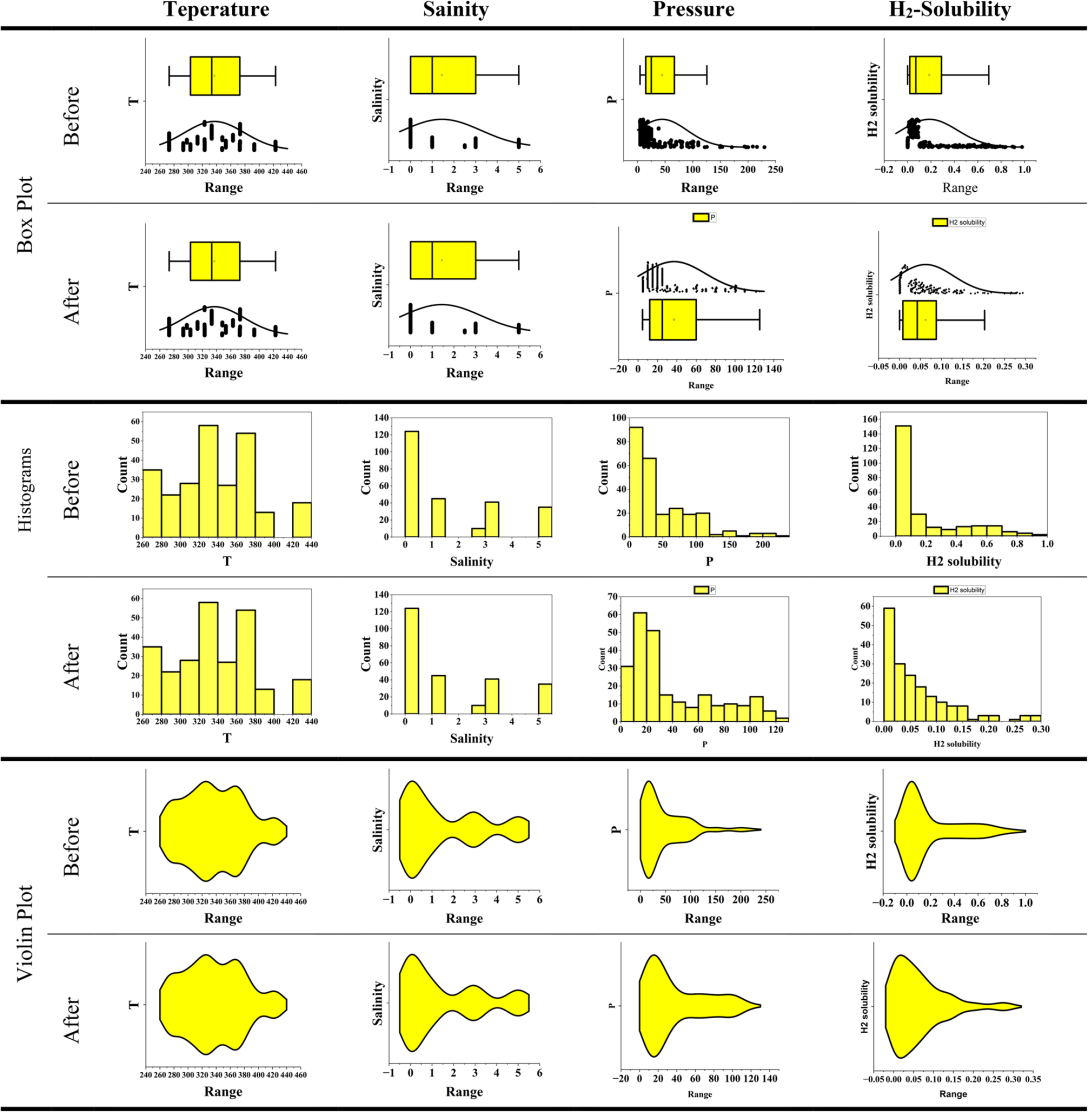
Specialized analysis of input data (Before and After outlier removal ). Box plots, Histograms, and Violin plots.

**Fig. 4:**
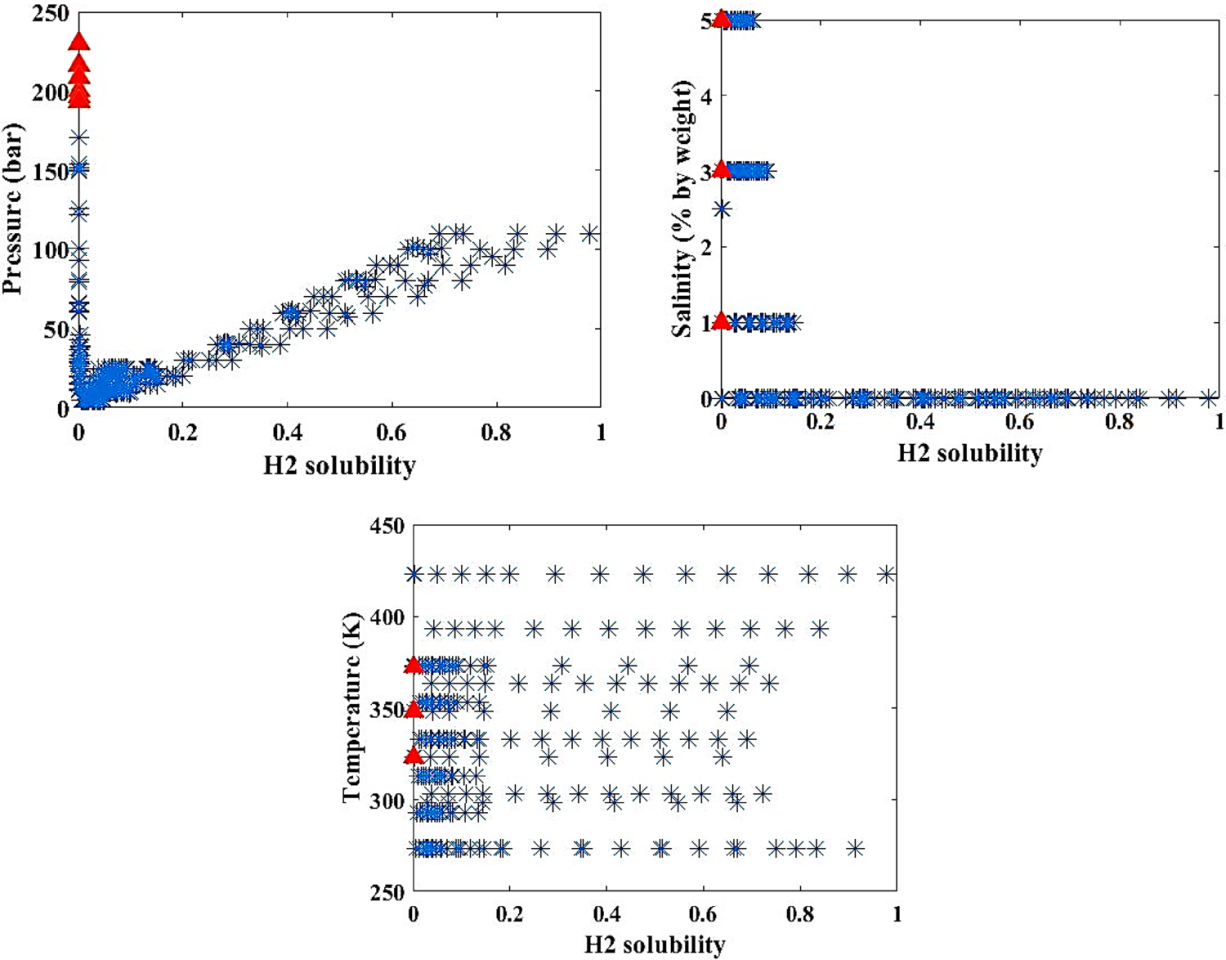
Outlier removal using the Gaussian method.

In this paper, the method of outlier removal using the Gaussian approach has been employed to enhance data quality. To demonstrate and visualize the improvement in data quality, various tools such as histograms, heat maps, box plots, and violin plots have been used. The process of this study is divided into three main stages:

1. Analysis of the data (Figs. 2 and 3).
2. Identification and visualization of outliers (where the red triangle represents the outliers and the “*” symbol indicates the remaining consistent data) (Fig. 4).

The statistical analysis confirms that temperature and pressure are among the most influential parameters affecting hydrogen solubility, as demonstrated by the trends observed in the box plots and regression plots.

The Gaussian Removal method, or outlier removal using the Gaussian distribution, is a statistical technique used to identify and remove outliers in a dataset. This method is based on the assumption that the data follows a normal distribution (Gaussian distribution), and data points that deviate significantly from this distribution are identified as outliers.

The process of the Gaussian Removal method consists of three main steps. First, the mean $\:\left(\mu\:\right)$ and standard deviation $\:\left(\sigma\:\right)$ of the dataset are calculated to identify outliers. Then, data points are identified as outliers if their distance from the mean is greater than one or more times the standard deviation. This distance is usually defined as $\:\mu\:\pm\:k\alpha\:$, where $\:k$ is a constant value that determines the sensitivity to outliers. Finally, data points that fall outside this range are identified as outliers and removed.

**Formulas for calculating mean and standard deviation**:

3 $$\:\mu\:=\frac{1}{n}\sum\:_{i=1}^{n}{x}_{i}$$

4 $$\:\sigma\:=\sqrt{\frac{1}{n}\sum\:_{i=1}^{n}{\left({x}_{i}-\mu\:\right)}^{2}}$$

Where $\:{x}_{i}$ are the data points, $\:n$ is the number of data points, and $\:\mu\:$ is the mean of the data.

**Gaussian removal equation**:

Data points identified as outliers follow the following equation:

5 $$\:P\left(x\right)=\frac{1}{\sigma\:\sqrt{2\pi\:}}exp\left(-\frac{{\left(x-\mu\:\right)}^{2}}{{2\sigma\:}^{2}}\right)$$

Where $\:P\left(x\right)$ is the probability density for data point $\:x$, $\:\mu\:$ is the mean of the data, $\:\sigma\:$ is the standard deviation of the data and $\:x$ are the data points.

Data points with very low probability values (i.e., significantly distant from the mean) are identified as outliers and removed.

### Machine learning methods

#### Bayesian linear regression

**Fig. 5:**
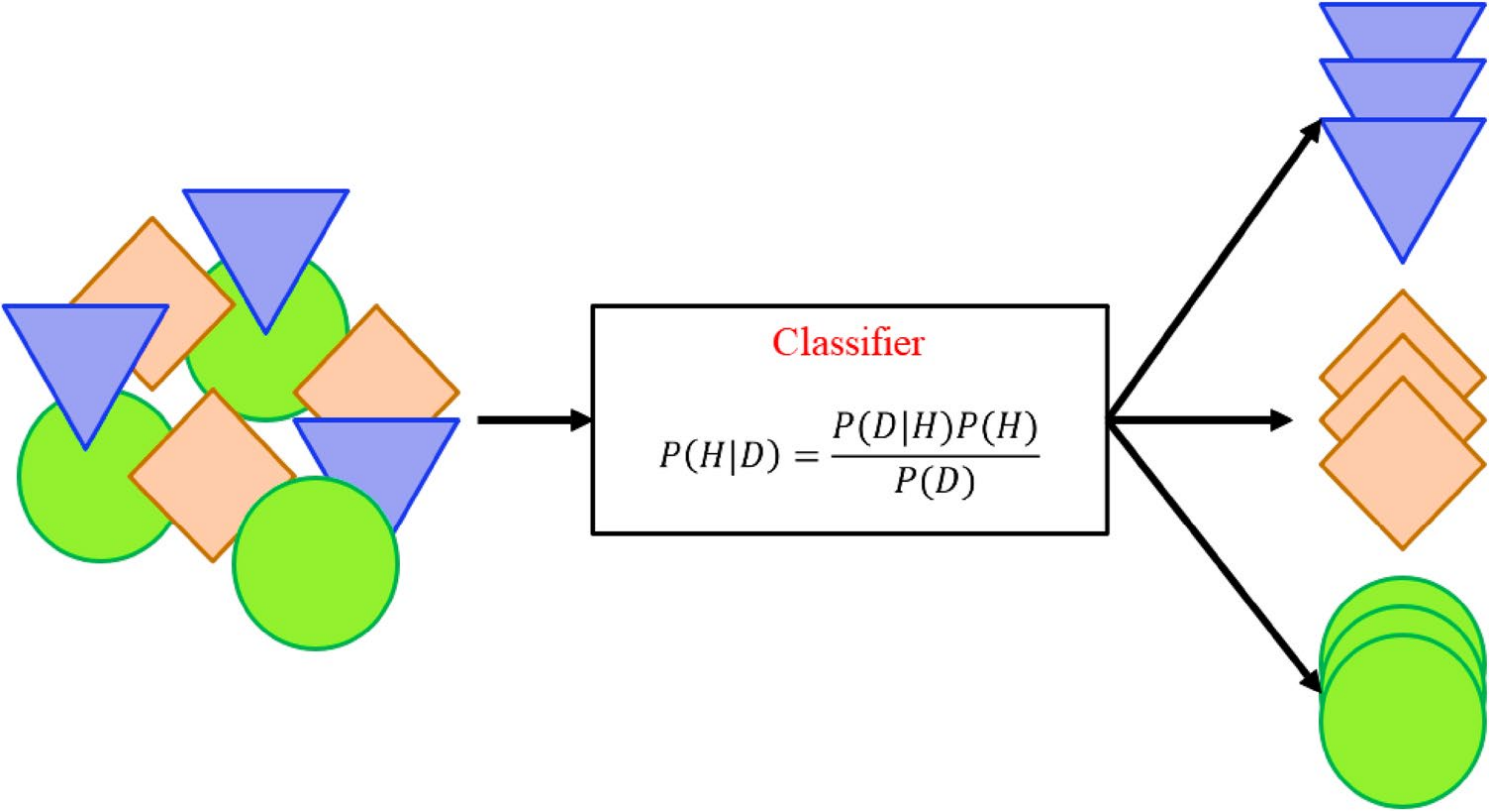
Bayesian algorithm.

Bayesian Linear Regression was employed to incorporate prior distributions over the model parameters, enabling regularization and uncertainty quantification during training. The Bayesian method is one of the fundamental approaches in machine learning, utilizing Bayesian probability principles for modeling and learning from data. This method combines prior knowledge and evidence from data. The core foundation of this approach is Bayes’ Theorem, which establishes a relationship between conditional probabilities (Fig. 5). Bayes’ Theorem is expressed as:

6 $$\:P\left(H|D\right)=\frac{P\left(D|H\right)P\left(H\right)}{P\left(D\right)}$$

In this equation, $\:P\left(H|D\right)$ represents the posterior probability, which indicates the likelihood of hypothesis $\:H$ given the evidence $\:D$. $\:P\left(D|H\right)$ is the likelihood, showing how probable the data $\:D$ is under the assumption that hypothesis $\:H$ is true. $\:P\left(H\right)$ is the prior probability, representing initial knowledge about hypothesis $\:H$, and $\:P\left(D\right)$ is the marginal likelihood, acting as a normalizing factor.

In machine learning, $\:H$ typically denotes the model or its parameters, while $\:D$ represents the training data. The goal is to estimate the posterior probability $\:P\left(D|H\right)$ to learn the model or its parameters. Bayesian methods are broadly categorized into parametric Bayesian learning and non-parametric Bayesian learning. In parametric learning, the model parameters are assumed to be fixed but unknown. For instance, if $\:\theta\:$ represents the model parameters, the posterior distribution is expressed as:

7 $$\:P\left(\theta\:|D\right)=\frac{P\left(D|\theta\:\right)P\left(\theta\:\right)}{P\left(D\right)}$$

In contrast, non-parametric Bayesian learning is employed when the number of parameters or the model structure is unknown. This approach is commonly used in models like Gaussian Processes or Bayesian clustering, where model complexity adjusts automatically based on the data.

One of the key applications of Bayesian methods in machine learning is prediction. Predictions are made using the posterior expectation. For example, the prediction of $\:{y}^{*}$ for a new data point $\:{x}^{*}$is calculated as:

8 $$\:P\left({y}^{*}|{x}^{*},D\right)=\int\:P\left({y}^{*}|{x}^{*},\theta\:\right)P\left(D|\theta\:\right)d\theta\:$$

Here, $\:P\left({y}^{*}|{x}^{*},\theta\:\right)$ is the predictive probability of the output $\:{y}^{*}$ given the input $\:{x}^{*}$ and parameters $\:\theta\:$, and $\:P\left(D|\theta\:\right)$ represents the posterior distribution of the parameters. This integral is often computed using methods such as Monte Carlo sampling (MCMC) or other approximation techniques.

Bayesian methods are applied in various models, such as the Naive Bayes Classifier and Bayesian Networks. In the Naive Bayes Classifier, it is assumed that the features are independent of each other, and the probability of a class $\:C$ given features $\:{x}_{1},\:{x}_{2},\:\dots\:,\:{x}_{n}$ is calculated as:

9 $$\:P\left(C|{x}_{1},\:{x}_{2},\:\dots\:,\:{x}_{n}\right)\propto\:P\left(C\right)\prod\:_{i=1}^{n}P\left({x}_{i}|C\right)$$

In contrast, Bayesian Networks utilize causal relationships between variables. In these models, nodes represent variables, and edges denote probabilistic dependencies among them.

The Bayesian method has several advantages and disadvantages. Its benefits include the ability to combine prior knowledge with new data, providing probabilistic distributions rather than deterministic values, and its applicability in scenarios with limited data. However, its primary drawbacks include computational complexity and sensitivity to the choice of the prior distribution.

#### Linear regression

Linear Regression is a foundational and widely used supervised machine learning algorithm. It models the relationship between independent variables (features) and a dependent variable (target) by fitting a linear equation to the observed data. The primary objective is to predict the value of the dependent variable based on the given independent variables. The general mathematical representation of linear regression is as follows:

10 $$\:y={\beta\:}_{0}+{\beta\:}_{1}{x}_{1}+{\beta\:}_{2}{x}_{2}+\dots\:+{\beta\:}_{p}{x}_{p}+ϵ$$

Where, $\:y$ is the dependent variable, $\:{x}_{1},{x}_{2},\:\dots\:,\:{x}_{p}\:$are the dependent variable, $\:{\beta\:}_{0}$ is the intercept, $\:{\beta\:}_{1},{\beta\:}_{2},\:\dots\:,\:{\beta\:}_{p}$ are the coefficients, and $\:ϵ$ is the error term. In matrix form, it can be written as:

11 $$\:y = X\beta \: + \in$$

Here, $\:y,\:X,\:\beta \:,\:and\: \in$ are matrices and vectors representing the data, coefficients, and errors. The main goal of linear regression is to minimize the error between the predicted and observed values, which is typically measured using the Mean Squared Error (MSE). The formula for MSE is:

12 $$\:MSE=\frac{1}{n}\sum\:_{i=1}^{n}{\left({y}_{i}-{\widehat{y}}_{i}\right)}^{2}$$

Where, $\:{y}_{i}$ represents the actual values, and $\:{\widehat{y}}_{i}$ represents the predicted values.

The parameters of the model $\:\left(\beta\:\right)$ are estimated using the Ordinary Least Squares (OLS) method. The OLS solution is derived as:

13 $$\:\beta\:={\left({X}^{T}X\right)}^{-1}{X}^{T}y$$

Once the model is trained, predictions for new data points are made using the following formula:

14 $$\:\widehat{y}=x\bullet\:\beta\:$$

Here, $\:x$ is the feature vector of the new data point.

#### Artificial neural network

**Fig. 6:**
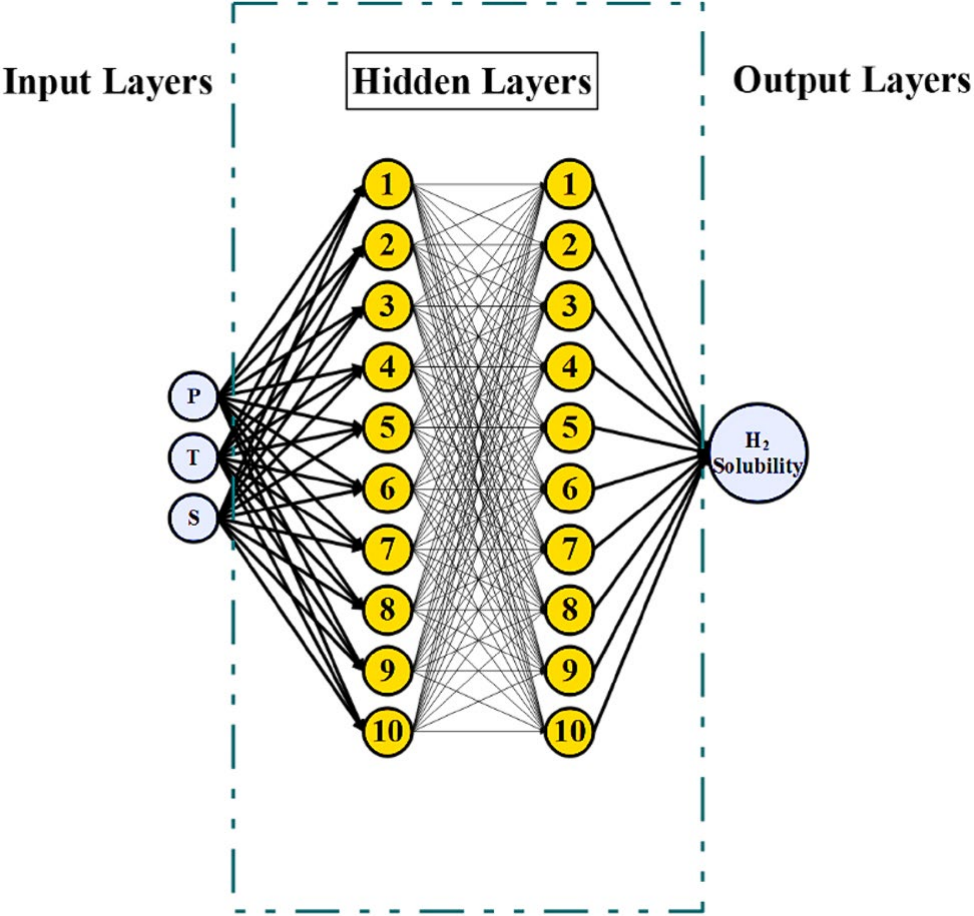
ANN algorithm.

Artificial Neural Networks (ANNs) are a foundational method in machine learning, modeled after the structure and operation of biological neural networks. ANNs are used to recognize patterns, classify data, make predictions, and model intricate relationships between inputs and outputs. They are composed of interconnected layers of nodes (neurons), each transforming input data and passing it to the next layer. The architecture of an ANN consists of three primary layers: the input layer, hidden layers, and the output layer. The input layer receives raw data, with each neuron corresponding to a single feature of the dataset. The hidden layers process the input data through transformations, and the output layer provides the final results, such as classifications or predictions (Fig. 6).

For a single neuron in a hidden or output layer, the pre-activation value $\:z$ is calculated as:

15 $$\:z=\sum\:_{i=1}^{n}{w}_{i}{x}_{i}+b$$

Here, $\:{x}_{i}$ inputs to the neuron, $\:{w}_{i}$ weights associated with the inputs, $\:b$ bias term, and $\:z$ pre-activation value.

#### Support vector machine

Support Vector Machines (SVMs) are a robust supervised machine learning algorithm commonly used for classification, regression, and outlier detection. They are particularly effective in high-dimensional spaces and are capable of handling both linear and nonlinear classification tasks. The primary objective of an SVM is to identify a hyperplane that effectively separates different classes of data points within the feature space.

The central idea of SVM is to determine a hyperplane that maximizes the margin between two classes. This margin is defined as the distance between the hyperplane and the nearest data points from each class, known as support vectors. For a training dataset represented as $\:\left\{\left({x}_{i},\:{y}_{i}\right)\right\}$, where $\:{x}_{i}$ is the feature vector and $\:{y}_{i}\in\:\left\{-1,\:+1\right\}$ is the class label, the hyperplane is mathematically defined as:

16 $$\:{w}^{T}x+b=0$$

Where, $\:w$ represents the weight vector, $\:x$ is the input feature vector, and $\:b$ is the bias term. The hyperplane acts as the decision boundary, while the support vectors are the data points closest to this boundary.

In scenarios where the data is linearly separable, SVM seeks the optimal hyperplane that maximizes the margin between the two classes. The equations governing the boundary of the margin are:

17 $$\:{w}^{T}{x}_{i}+b=+1\:\:\:\:\:for\:{y}_{i}=+1$$

18 $$\:{w}^{T}{x}_{i}+b=-1\:\:\:\:\:for\:{y}_{i}=-1$$

This approach ensures that the hyperplane achieves maximum separation while maintaining the closest points (support vectors) at the boundary of the margin.

#### Least squares boosting

Least Squares Boosting (LSBoost) is a machine learning method that integrates boosting with least squares regression to improve the accuracy of predictions. It is mainly used for regression tasks but can also be adapted to classification problems. LSBoost enhances traditional boosting techniques by focusing on minimizing the least squares error of the model. The process begins with an initial simple model, typically the mean of the target values. In each iteration of boosting, residuals are computed by calculating the difference between the actual values and the model’s current predictions. A new weak learner, usually a decision tree, is then trained to predict these residuals, aiming to minimize the least squares error. The model is updated by adding the scaled predictions of this weak learner, which are adjusted by a learning rate. After a predefined number of iterations or when the model’s performance stabilizes, the final model is constructed by combining the predictions of all weak learners.

The initial prediction is computed as:

19 $$\:{\widehat{y}}_{0}=\frac{1}{N}\sum\:_{i=1}^{N}{y}_{i}$$

Where, $\:{y}_{i}$ is the actual target value for the $\:{i}^{th}$ instance and $\:N$ is the total number of instances.

At iteration $\:m$, the residuals are calculated as:

20 $$\:{r}_{i}\left(m\right)={y}_{i}-{\widehat{y}}_{i}\left(m\right)$$

Where $\:{\widehat{y}}_{i}\left(m\right)$ represents the prediction for the $\:{i}^{th}$ instance at iteration $\:m$.

A weak learner is then fit to these residuals, aiming to minimize the least squares error, expressed as:

21 $$\:min\sum\:_{i=1}^{N}{\left({r}_{i}\left(m\right)-{f}_{m}\left({x}_{i}\right)\right)}^{2}$$

Where $\:{f}_{m}\left({x}_{i}\right)$ is the prediction of the weak learner for the $\:{i}^{th}$ instance.

The model is updated by adding the predictions of the weak learner, scaled by a learning rate $\:\alpha\:$:

22 $$\:{\widehat{y}}_{i}\left(m+1\right)={\widehat{y}}_{i}\left(m\right)+\alpha\:{f}_{m}\left({x}_{i}\right)$$

Where $\:\alpha\:$ is the learning rate (also known as the shrinkage parameter).

After $\:M$ iterations, the final prediction is given by:

23 $$\:{\widehat{y}}_{i}={\widehat{y}}_{0}+\sum\:_{m=1}^{M}\alpha\:{f}_{m}\left({x}_{i}\right)$$

#### Random forest

**Fig. 7:**
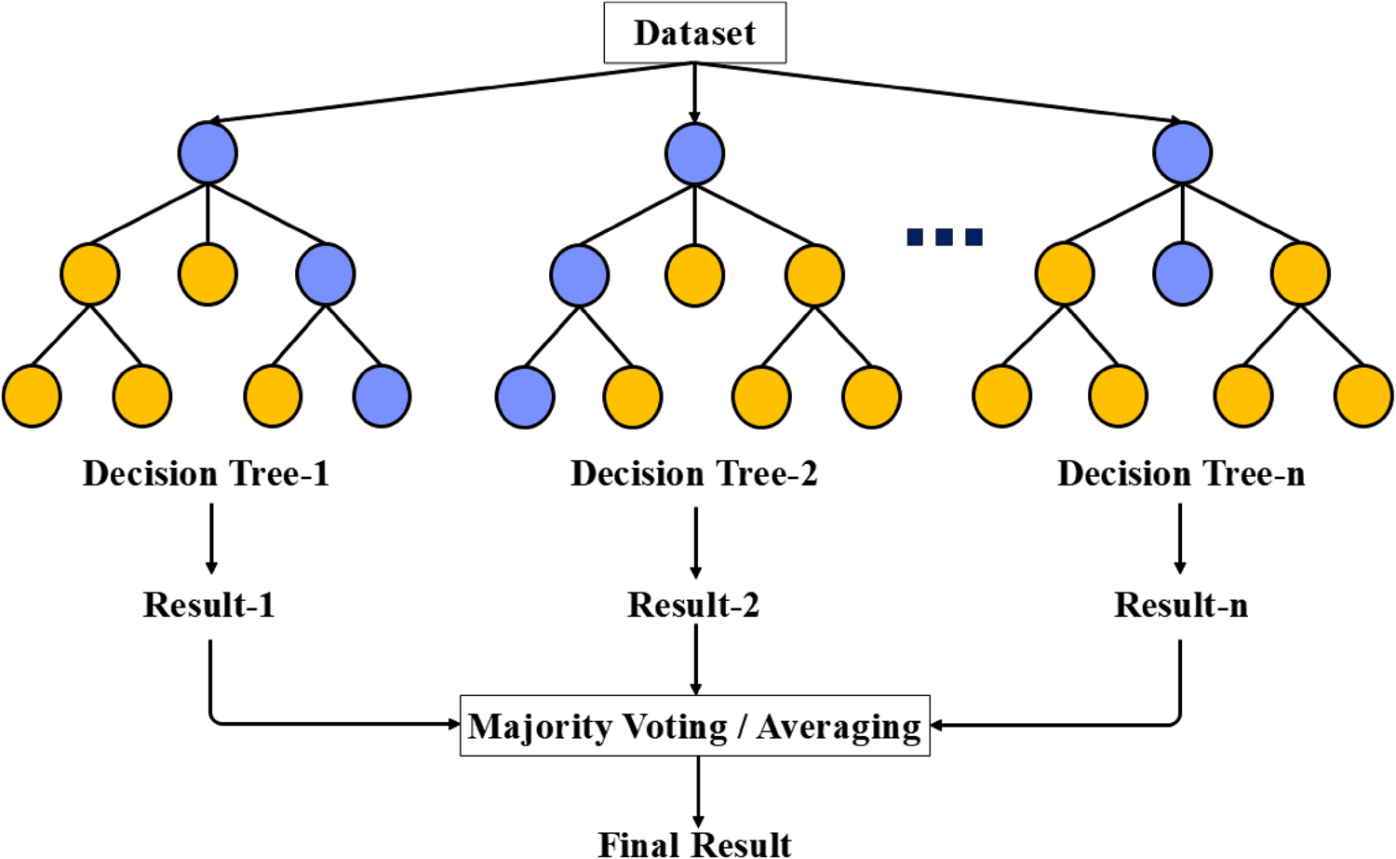
RF algorithm.

Random Forest (RF) is a widely used machine learning technique that excels in classification and regression tasks, especially when working with large and complex datasets. Its ability to reduce variance and prevent overfitting makes it a popular choice. RF is an ensemble method that combines multiple decision trees, each trained independently on a random subset of the data. The final result is obtained by aggregating the predictions from these individual trees (Fig. 7).

The process of the RF algorithm starts by generating random samples from the training data through bootstrap sampling (sampling with replacement). Each decision tree is trained on a different random subset of the data. To introduce diversity among the trees, a random selection of features is made at each tree node. When predicting, the results from all trees are combined. For classification tasks, the final prediction is determined by majority voting, whereas for regression tasks, the predictions are averaged.

In classification, the final prediction for a new sample $\:x$ is computed using the following formula, where $\:{T}_{1},\:\:{T}_{2},\:\dots\:,\:\:{T}_{n}$ represent the decision trees and $\:{C}_{1},\:\:{C}_{2},\:\dots\:,\:\:{C}_{n}$ are the possible classes:

24 $$\:Prediction\:\left(x\right)=\text{arg}max\left(\sum\:_{j=1}^{n}I\left({T}_{j}\left(x\right)={C}_{i}\right)\right)$$

Here, $\:I$ is an indicator function that equals 1 if $\:{T}_{j}\left(x\right)$ equals $\:{C}_{i}$ and 0 otherwise.

For regression tasks, the final prediction for a new sample $\:x$ is calculated as the average of the predictions from all the decision trees:

25 $$\:Prediction\:\left(x\right)=\frac{1}{n}\sum\:_{j=1}^{n}{T}_{j}\left(x\right)$$

Where $\:{T}_{j}\left(x\right)$ is the predicted value from decision tree $\:{T}_{j}$ for the sample $\:x$.

## Results and discussion

### Data division into training and testing sets

At the beginning of this study, all collected input parameters were thoroughly and comprehensively analyzed. The input parameters, including Salinity (% by weight), Temperature (K), and Pressure (bar), were then selected for performing calculations using machine learning algorithms in MATLAB software. Subsequently, the input data was divided into two separate sets: one for training and the other for testing, in order to evaluate the performance of the algorithms. The proposed algorithms were executed for predicting Hydrogen Solubility (mole fraction) based on various training-to-testing data ratios, and the accuracy of each method was computed and presented graphically.

The random selection of input data based on these ratios can significantly impact the final accuracy of the algorithms. Accordingly, each method was evaluated through ten independent runs, and the average R^2^ values obtained from these runs were reported as the final results. This evaluation approach provides an accurate representation of the performance and precision of each algorithm.

Table 3 reports the final R^2^ values for each layer within the range of 0.1 to 0.9. These values are independently calculated for each machine learning method and presented for the testing, training, and training/testing combined datasets. In fact, based on the results obtained from the training/testing rows, optimal models can be selected.

**Table 3:** Final R2 values for each layer across ML Methods.

	Precision	*R*^2^ - Bayesian	*R*^2^ - LR	*R*^2^ - ANN	*R*^2^ - SVM	*R*^2^ - LSBoost	*R*^2^ - RF
Test	0.1	0.6813	0.4841	0.6995	0.6569	0.6162	0.7335
	0.2	0.6807	0.6809	0.9088	0.8731	0.8372	0.9348
	0.3	0.6798	0.6799	0.9185	0.8855	0.8752	0.9409
	0.4	0.6739	0.6743	0.9215	0.9167	0.8549	0.9432
	0.5	0.6633	0.6637	0.9216	0.9207	0.8840	0.9497
	0.6	0.6785	0.6786	0.9201	0.9149	0.8929	0.9577
	0.7	0.6679	0.6681	0.9259	0.9248	0.9090	0.9632
	0.8	0.6851	0.6852	0.9245	0.9220	0.9195	0.9709
	0.9	0.6838	0.6838	0.9261	0.9404	0.9207	0.9806
Train	0.1	0.6680	0.6695	0.7840	0.7470	0.7074	0.7994
	0.2	0.8849	0.8849	0.9921	0.9598	0.9597	1.0000
	0.3	0.8559	0.8559	0.9847	0.9569	0.9450	0.9971
	0.4	0.8310	0.8310	0.9842	0.9634	0.9828	0.9975
	0.5	0.8593	0.8593	0.9779	0.9729	0.9554	0.9997
	0.6	0.8249	0.8249	0.9760	0.9612	0.9556	0.9971
	0.7	0.7975	0.7975	0.9699	0.9551	0.9405	0.9976
	0.8	0.7557	0.7557	0.9652	0.9668	0.9485	0.9942
	0.9	0.7401	0.7401	0.9620	0.9483	0.9437	0.9915
Test /Train (All)	0.1	0.6746	0.5693	0.7406	0.7005	0.6602	0.7658
	0.2	0.7761	0.7763	0.9496	0.9154	0.8963	0.9668
	0.3	0.7628	0.7629	0.9510	0.9205	0.9094	0.9686
	0.4	0.7483	0.7485	0.9523	0.9398	0.9166	0.9699
	0.5	0.7549	0.7552	0.9494	0.9464	0.9190	0.9744
	0.6	0.7481	0.7482	0.9476	0.9378	0.9237	0.9772
	0.7	0.7298	0.7299	0.9477	0.9398	0.9246	0.9802
	0.8	0.7196	0.7196	0.9446	0.9441	0.9339	0.9825
	0.9	0.7114	0.7114	0.9439	0.9443	0.9321	0.9860

The main criterion for selecting the final results is based on the Test/Train ratio (All). Accordingly, the maximum value of each method is selected, and the corresponding regression plots are then drawn.

**Fig. 8:**
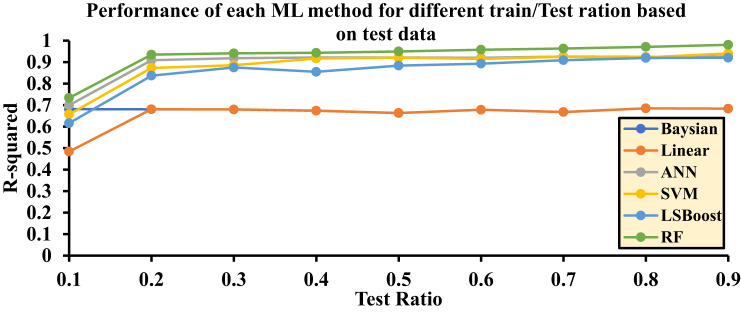
Accuracy of different ML methods in different percentages of training-to-test data based on test data.

**Fig. 9:**
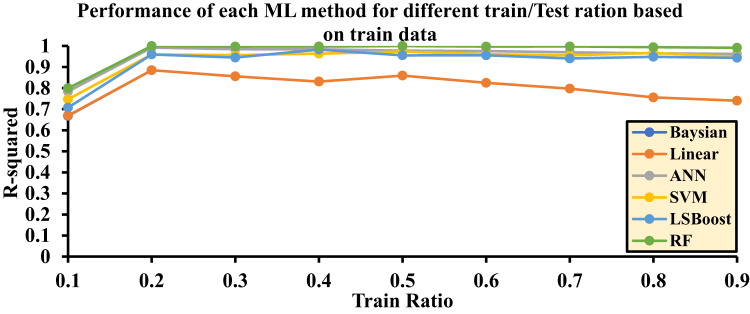
Accuracy of different ML methods in different percentages of training-to-test data based on train data.

**Fig. 10:**
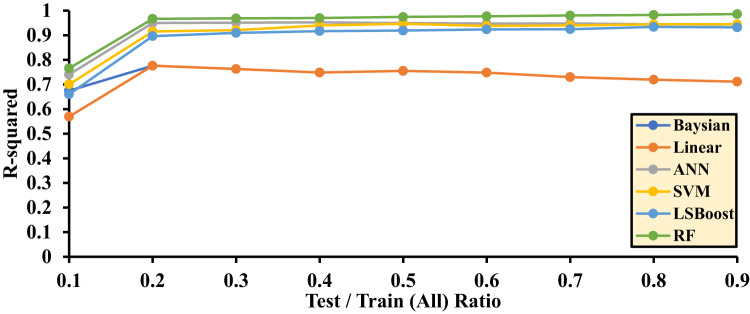
Accuracy of different ML methods in different percentages of training-to-test data.

Evaluation of the optimal performance of Hydrogen solubility for each ML method:

Figure 8 is drawn based on the test data, and Fig. 9 is drawn based on the training data for Hydrogen solubility, with the accuracy of each method being evaluated. The final decision and selection of the best R^2^ value are referenced in the Test/Train Ratio section (Fig. 10).

Based on the results related to hydrogen solubility presented in Fig. 10, the highest accuracy is achieved when the training-to-testing data ratio in the algorithms is as follows: 0.2 for the Bayesian algorithm, 0.2 for the LR algorithm, 0.4 for the ANN algorithm, 0.5 for the SVM algorithm, 0.8 for the LSBoost algorithm, and 0.9 for the RF algorithm.

The dataset comprising 992 cleaned samples was split into training and testing subsets based on the optimal ratios identified for each machine learning algorithm. The following Table 4 presents the corresponding number of samples used for training and testing in each case.

**Table 4:** Data split configuration (train and test sizes) based on the optimal ratio for each ML algorithm.

Algorithm	Train ratio (%)	Test ratio (%)	Train size	Test size
Bayesian	20%	80%	198	794
LR	20%	80%	198	794
ANN	40%	60%	397	595
SVM	50%	50%	496	496
LSBoost	80%	20%	794	198
RF	90%	10%	893	99

Table 5 contains the key control parameters for various machine learning algorithms, which were carefully tuned and optimized to ensure the accuracy and reliability of the results. It includes the values and descriptions of relevant parameters for each algorithm, such as SVM, RF, ANN, LSBoost, Bayesian Method, and LR. These settings were selected based on preliminary testing, cross-validation, and best practices reported in the literature to optimize the performance of each algorithm.

**Table 5:** Control parameters for each Algorithm.

Algorithm	Control Parameter	Value/description
SVM	Kernel Function	Radial Basis Function (RBF) kernel
	Regularization Parameter	10
	Tolerance	0.001
RF	Number of Trees	100 decision trees
	Maximum Depth	10
	Minimum Samples Split	2
	Bootstrap Sampling	Enabled (random sampling with replacement)
ANN	Number of Layers	3-layer architecture (1 input, 1 hidden with 10 neurons, 1 output)
	Activation Function	ReLU for hidden layer, linear for output layer
	Learning Rate	0.01
	Epochs	500 training iterations
	Batch Size	32
LSBoost	Number of Iterations	50 boosting iterations
	Learning Rate (Shrinkage Parameter)	0.1
	Base Learner	Decision trees with a maximum depth of 3
Bayesian	Prior Distribution	Gaussian prior
	Sampling Algorithm	Markov Chain Monte Carlo (MCMC)
	Burn-in Period	100 iterations
	Number of Samples	1000 samples
LR	Regularization	Ridge regression with L2 regularization (α = 0.01)
	Optimization Algorithm	Ordinary Least Squares (OLS)
	Tolerance	1e-4 for convergence

### Performance of each method in the training and testing phases

The results obtained from regression analyses and R^2^ values are presented in graphical plots, where the data from both the training (Train) and testing (Test) sets are displayed simultaneously. These plots clearly show the model’s fit to the data with the final R^2^ value, accurately indicating the model’s performance. This method is particularly useful for evaluating the accuracy and performance of the model in predicting test data and also facilitates the comparison of model performance in both the training and testing datasets.

**Fig. 11:**
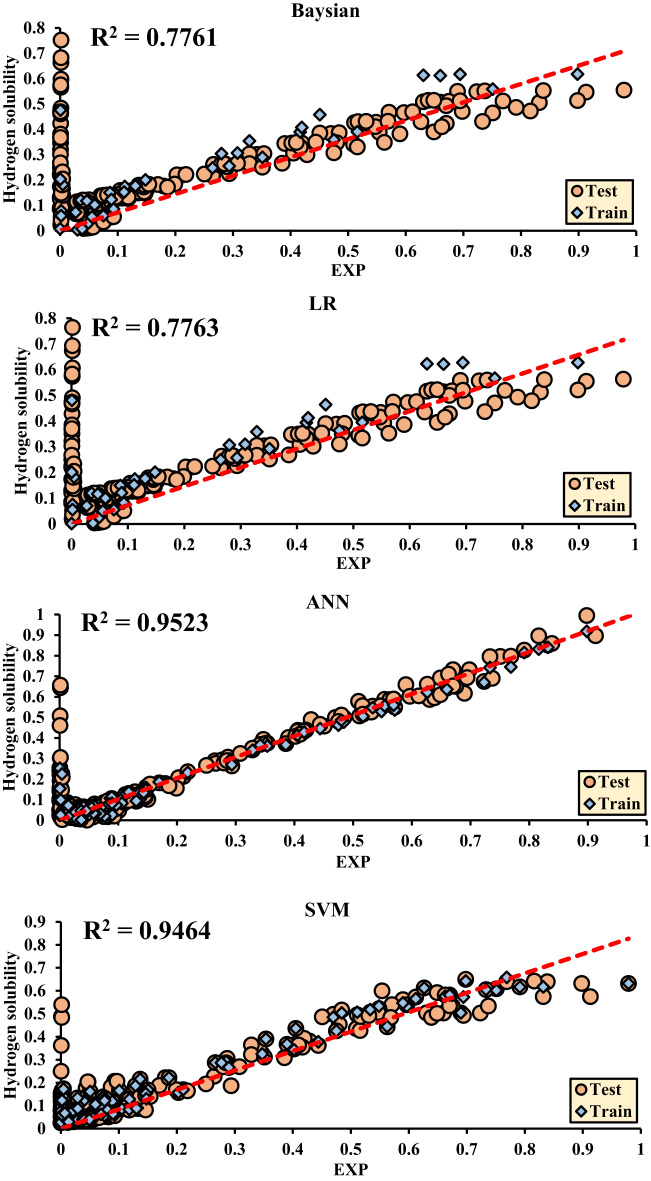

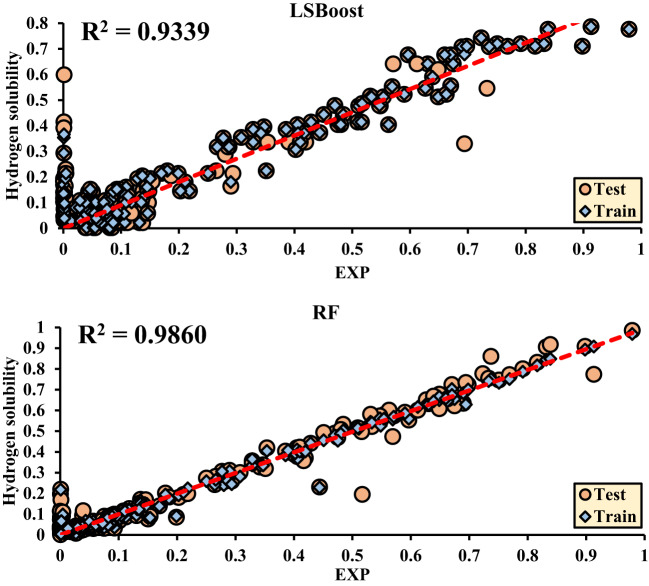
shows the R^2^ values obtained from hydrogen solubility.

Figure 11. Regression of Different ML Methods for Hydrogen solubility.

### Statistical criteria in measuring the accuracy of machine learning methods

In conclusion, the effectiveness and performance of the methods discussed in this article have been evaluated and compared using statistical metrics such as the correlation coefficient and mean relative error. The following equations describe the calculation process for each of these statistical measures. Table 6 presents the performance values for each method.

Root Mean Square Error (RMSE) is a statistical tool used to evaluate the effectiveness of predictive models. It measures the degree of difference between the predicted values of the model and the actual values. RMSE is commonly used to assess the accuracy of the model by calculating the average squared error. The RMSE value can range from zero to infinity, with values closer to zero indicating better model accuracy.

26 $$\:RMSE=\sqrt{\frac{1}{n}\sum\:_{i=1}^{n}{\left({y}_{i}-{\widehat{y}}_{i}\right)}^{2}}$$

Mean Square Error (MSE) is another statistical measure used to evaluate prediction models. MSE quantifies the difference between the predicted values of the model and the actual values. Similar to RMSE, MSE is based on the squared errors. Like RMSE, the MSE value ranges from zero to infinity, with values closer to zero signifying higher accuracy of the model.

27 $$\:MSE=\frac{1}{n}\sum\:_{i=1}^{n}{\left({y}_{i}-{\widehat{y}}_{i}\right)}^{2}$$

The Mean Absolute Deviation (MAD) is a metric used to assess the precision of predictive models by averaging the absolute differences between the actual and predicted values. A lower MAD value indicates better model accuracy, and a MAD of zero signifies perfect correspondence between the model’s predictions and the real data. This metric is especially valuable for comparing the performance of different models and refining them for optimal results.

28 $$\:MAD=\frac{1}{n}\sum\:_{i=1}^{n}\left|{y}_{i}-{\widehat{y}}_{i}\right|$$

In this formula, $\:{y}_{i}$ represents the actual value for sample $\:i$, and $\:{\widehat{y}}_{i}$ is the predicted value for the same sample. $\:n$ denotes the total number of samples. This formula allows us to account for both positive and negative errors as absolute values, meaning all errors are calculated as positive values, which reduces the impact of large errors.

R^2^ is a statistical measure used to evaluate the effectiveness of prediction models and is widely recognized as a key criterion for model assessment. Also known as the coefficient of determination, R^2^ represents the fraction of the variance in the dependent variable that can be explained by the independent variables in the model’s predictions.

A value of R^2^ close to 1 indicates that the model has successfully explained most of the variations in the dependent variable, with the predicted values being highly accurate. On the other hand, an R^2^ value close to 0 suggests that the model has failed to account for much of the variation in the dependent variable, leading to predicted values that deviate significantly from the actual outcomes.

29 $${R^2}=1 - \frac{{\mathop \sum \nolimits_{{i=1}}^{N} {{\left( {y_{i}^{{Pred}} - y_{i}^{{exp}}} \right)}^2}}}{{\mathop \sum \nolimits_{{i=1}}^{N} \left( {y_{i}^{{Pred}} - average(y_{i}^{{exp}}} \right){)^2}}}$$

**Table 6:** Plotted error values of ML methods.

	Method	*R*^2^ (Test)	*R*^2^ (Train)	*R*^2^ (All)	MAD (Test)	MAD (Train)	MSE (Test)	MSE (Train)	RMSE (Test)	RMSE (Train)
Hydrogen solubility	Bayesian	0.6807	0.8849	0.7761	0.122	0.069	0.032	0.11	0.18	0.106
	LR	0.6810	0.8849	0.7763	0.122	0.069	0.033	0.011	0.18	0.106
	ANN	0.9215	0.9842	0.9523	0.048	0.026	0.01	0.002	0.101	0.046
	SVM	0.9207	0.9729	0.9464	0.064	0.05	0.01	0.005	0.102	0.072
	LSBoost	0.9195	0.9485	0.9339	0.065	0.059	0.009	0.006	0.095	0.08
	RF	0.9810	0.9915	0.9860	0.027	0.018	0.002	0.001	0.048	0.032

**Fig. 12:**
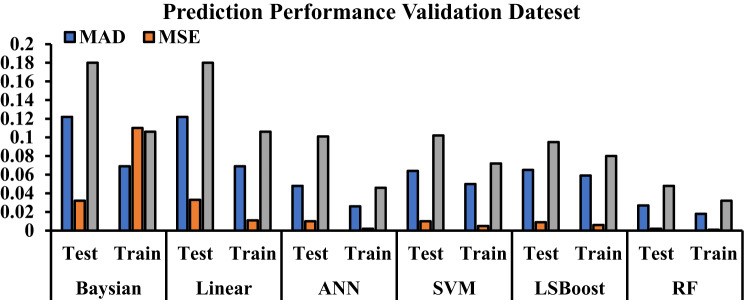
The error of each machine learning method.

Figure 12 visually presents the error data from Table 6, allowing for a straightforward comparison of the methods.

Among the evaluated machine learning models, several—including ANN (R^2^ = 0.9523), SVM (R^2^ = 0.9464), and LSBoost (R^2^ = 0.9339)—exhibited strong and closely matched predictive capabilities. However, the Random Forest (RF) model slightly outperformed the others, achieving the highest R^2^ value of 0.9860, along with the lowest RMSE (0.048) and MAD (0.027). These results suggest that although multiple models were effective, RF offered a marginal yet meaningful advantage in capturing the complex relationships between the input variables and hydrogen solubility.

Table 7 presents the results of the best method (RF) for predicting the hydrogen solubility characteristics. This table reports various metrics for evaluating the model’s accuracy, including the following:

**Table 7:** Best results for each of the hydrogen solubility.

Best Method	*R*^2^ (Test)	*R*^2^ (Train)	*R*^2^ (All)	MAD (Test)	MAD (Train)	MSE (Test)	MSE (Train)	RMSE (Test)	RMSE (Train)
RF	0.9810	0.9915	0.9860	0.027	0.018	0.002	0.001	0.048	0.032

This table illustrates the performance of the RF method as the best approach for data analysis. Below, the various model evaluation criteria are explained:

**Coefficient of determination (R**^**2**^**)**:

- For test data (Test): A value of 0.9810 indicates very high prediction accuracy.
- For training data (Train): A value of 0.9915 shows that the model also performs very accurately during training.
- For all data (All): A value of 0.9860 reflects the overall balance and efficiency of the model.

**Mean absolute deviation (MAD)**:

- For test data (Test): A value of 0.027 indicates a low average difference between predicted and actual values.
- For training data (Train): A value of 0.018 demonstrates higher accuracy during the training phase.

**Mean squared error (MSE)**:

- For test data (Test): A value of 0.002 indicates a low level of error in predictions.
- For training data (Train): A value of 0.001 reflects even lower error during training.

**Root mean squared error (RMSE)**:

- For test data (Test): A value of 0.048 represents relatively low prediction error.
- For training data (Train): A value of 0.032 highlights the model’s very high accuracy on training data.

In addition to the statistical evaluation, the superior performance of the RF model can be attributed to its inherent capability to capture complex non-linear interactions and to effectively handle noisy or heterogeneous data. The prediction of hydrogen solubility in aqueous systems involves thermodynamic variables—particularly pressure and temperature—whose relationships with solubility are highly non-linear and system-dependent. Considering that the dataset includes measurements across a wide range of pressure and temperature conditions from diverse experimental sources, the ensemble-based structure of RF enables the modeling of intricate patterns without overfitting. Moreover, RF is known for its robustness to data irregularities and measurement uncertainties, making it especially suitable for applications involving multi-source scientific data. These characteristics explain why the RF model consistently outperformed the other algorithms in the current study.

## Conclusions

Hydrogen storage in subsurface environments presents a promising approach for reducing CO_2_ emissions, particularly in the oil and gas industry. However, a key challenge in this process is the solubility of hydrogen in saline aquifers, which significantly influences the efficiency and stability of storage reservoirs. The dissolution of hydrogen can alter reservoir pressure and affect the chemical composition of the storage medium, necessitating a comprehensive understanding of fluid dynamics under such conditions. This study highlights the critical role of hydrogen solubility in optimizing underground storage systems and underscores the need for advanced predictive techniques.

The application of machine learning models has demonstrated considerable potential in predicting hydrogen solubility under varying pressure, temperature, and salinity conditions. By leveraging advanced algorithms such as Bayesian methods, linear regression, random forest, artificial neural networks (ANN), support vector machines (SVM), and least squares boosting (LSBoost), this study provides accurate predictions of hydrogen solubility in saline aquifers. These models effectively capture complex nonlinear relationships between influential parameters, offering valuable insights for optimizing hydrogen storage strategies.

A total of 1020 data points were originally gathered from multiple literature sources. After identifying and removing outliers using the Gaussian-based approach, the dataset was refined to 992 reliable samples, which were then employed for training and evaluating the machine learning models. The analysis of pressure data, ranging from approximately 5 bar to over 200 bar, revealed critical trends using statistical visualization techniques such as box plots. Similarly, temperature data spanning from 273 K to 423 K were analyzed through violin and box plots, demonstrating the significant impact of thermal conditions on hydrogen solubility. Salinity data analysis indicated clustering around specific concentrations, such as 0–3 g/L, providing essential insights into the role of salinity in hydrogen dissolution behavior.

The findings of this study emphasize that pressure, temperature, and salinity exert a simultaneous and complex influence on hydrogen solubility in subsurface environments. The integration of machine learning techniques enhances predictive accuracy and contributes to the development of efficient hydrogen storage methodologies. Future research should focus on refining these models with expanded datasets and incorporating additional environmental parameters to further improve prediction accuracy and storage feasibility. These advancements will be instrumental in optimizing large-scale hydrogen storage and supporting the transition towards sustainable energy solutions.

The findings confirm that various machine learning models, especially ANN, SVM, and LSBoost, can effectively predict hydrogen solubility in saline systems. Nonetheless, the Random Forest algorithm demonstrated the most robust performance overall, providing slightly higher accuracy and lower error metrics. Its consistent results across training and testing sets highlight its reliability in modeling nonlinear behaviors related to thermodynamic parameters. These insights support the integration of RF and similar ensemble-based approaches into future predictive frameworks for hydrogen storage applications.

The results of each method based on R^2^ and RMSE:

- RF: The RF model achieved the highest accuracy with $\:{R}_{Test}^{2}$ = 0.9810, $\:{R}_{Train}^{2}$ = 0.9915, $\:{RMSE}_{Test}$ = 0.048, and $\:{RMSE}_{Train}$ = 0.032, making it the most effective method for predicting hydrogen solubility.
- ANN: $$\:{R}_{Test}^{2}$$ = 0.9215, $$\:{R}_{Train}^{2}$$ = 0.9842, $$\:{RMSE}_{Test}$$ = 0.101, and $$\:{RMSE}_{Train}$$ = 0.046.
- SVM: $$\:{R}_{Test}^{2}$$ = 0.9207, $$\:{R}_{Train}^{2}$$ = 0.9729, $$\:{RMSE}_{Test}$$ = 0.102, and $$\:{RMSE}_{Train}$$ = 0.072.
- LSBoost: $$\:{R}_{Test}^{2}$$ = 0.9195, $$\:{R}_{Train}^{2}$$ = 0.9485, $$\:{RMSE}_{Test}$$ = 0.095, and $$\:{RMSE}_{Train}$$ = 0.08.
- LR: $$\:{R}_{Test}^{2}$$ = 0.6810, $$\:{R}_{Train}^{2}$$ = 0.8849, $$\:{RMSE}_{Test}$$ = 0.18, and $$\:{RMSE}_{Train}$$ = 0.106.
- Bayesian: $$\:{R}_{Test}^{2}$$ = 0.6807, $$\:{R}_{Train}^{2}$$ = 0.8849, $$\:{RMSE}_{Test}$$ = 0.18, and $$\:{RMSE}_{Train}$$ = 0.106.

RF outperformed all other models, showing the highest accuracy and lowest error in both training and testing datasets.

## Data Availability

Data availabilityThe datasets used and/or analyzed during the current study available from the corresponding author on reasonable request.

## Abbreviations

ANN: artificial neural network
CNN: convolutional neural networks
CSA: coupled simulated annealing
DBN: deep belief network
EOR: enhanced oil recovery
GA: genetic algorithm
GO: growth optimization
GWO: grey wolf optimization
IQR: interquartile range
LR: linear regression
LSBoost: least squares boosting
LSSVM: least squares support vector machine
LSTM: long short-term memory
MAD: mean absolute deviation
MAE: mean absolute error
MARS: multivariate adaptive regression splines
MCMC: Markov chain monte Carlo
ML: machine learning
MSE: mean squared error
Naive Bayes: naive bayes classifier
OLS: ordinary least squares
PR: peng-robinson (equation of State)
PSO: particle swarm optimization
R^2^: coefficient of determination
RBF: radial basis function
RF: random forest
RK: Redlich-Kwong (equation of State)
RMSE: root mean square error
SRK: soave-Redlich-Kwong (equation of State)
SVM: support vector machines
UHS: underground hydrogen storage
ZJ: Zudkevitch-Joffe (Equation of State)
